# Targeting the Metastatic Bone Microenvironment by MicroRNAs

**DOI:** 10.3389/fendo.2018.00202

**Published:** 2018-04-27

**Authors:** Marie-Therese Haider, Hanna Taipaleenmäki

**Affiliations:** Molecular Skeletal Biology Laboratory, Department of Trauma, Hand and Reconstructive Surgery, University Medical Center Hamburg-Eppendorf, Hamburg, Germany

**Keywords:** breast cancer, bone metastases, microRNA, bone microenvironment, osteoclast, osteoblast

## Abstract

Bone metastases are a common and devastating feature of late-stage breast cancer. Metastatic bone disease is a consequence of disturbed bone remodeling due to pathological interactions between cancer cells and the bone microenvironment (BME). In the BME, breast cancer cells severely alter the balanced bone formation and bone resorption driven by osteoblasts and osteoclasts. The complex cellular cross talk in the BME is governed by secreted molecules, signaling pathways and epigenetic cues including non-coding RNAs. MicroRNAs (miRNAs) are small non-coding RNAs that reduce protein abundance and regulate several biological processes, including bone remodeling. Under pathological conditions, abnormal miRNA signaling contributes to the progression of diseases, such as bone metastasis. Recently miRNAs have been demonstrated to regulate several key drivers of bone metastasis. Furthermore, miRNAs are implicated as important regulators of cellular interactions within the metastatic BME. As a consequence, targeting the BME by miRNA delivery or antagonism has been reported to limit disease progression in experimental and preclinical conditions positioning miRNAs as emerging novel therapeutic tools in metastatic bone disease. This review will summarize our current understanding on the composition and function of the metastatic BME and discuss the recent advances how miRNAs can modulate pathological interactions in the bone environment.

## Introduction

Breast cancer is one of the most common malignancies in the world. Approximately 12% of women are diagnosed with breast cancer during their lifetime (1). After successful treatment of the primary tumor that often comprises surgery, adjuvant chemo- and radiation therapy, and the administration of anti-hormonal drugs, patients frequently suffer from distant metastases even decades after a disease-free interval (2). Bone is the most common site for breast cancer metastases, and approximately 70% of patients with advanced breast cancer suffer from osteolytic bone metastases (3). Osteolytic metastases are frequently associated with skeletal-related events (SREs), including fractures and spinal cord compression, that are often accompanied by severe pain and hypercalcemia (4).

**Figure 1:**
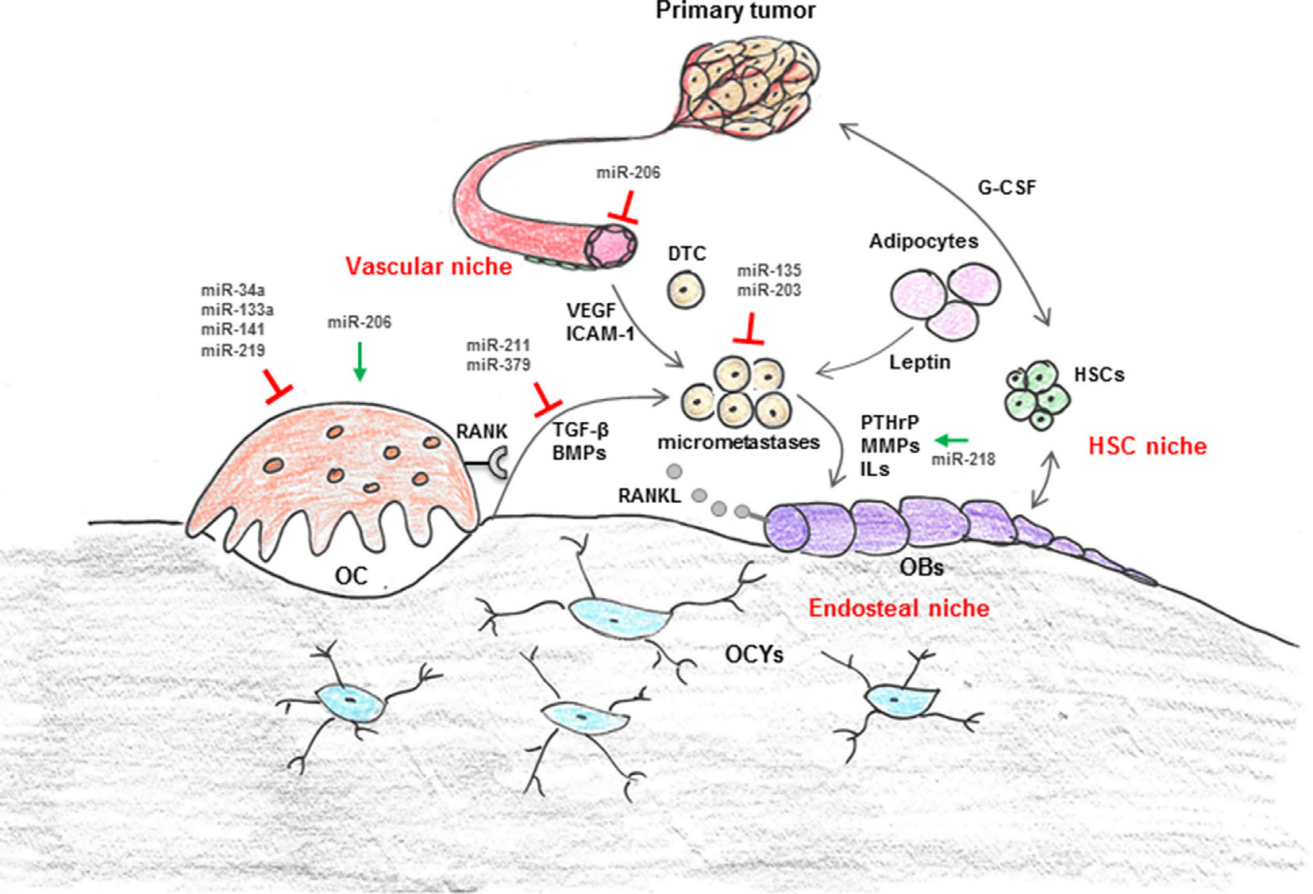
Regulation of cellular interaction in breast cancer bone metastasis by microRNA (miRNAs). The bone microenvironment (BME) is composed of cellular entities, including hematopoietic stem cells (HSC niche), osteoblasts (OBs), osteoclasts (OCs), and adipocytes (endosteal niche) as well as vascular endothelial cells and pericytes (vascular niche). These niches are suggested to control survival, dormancy, and growth of disseminated tumor cells (DTCs) through production of cytokines (i.e., leptin, G-CSF, VEGF, etc.) and intracellular signals in addition to cell-to-cell contact. In a physiological context, the highly coordinated cross talk between bone-forming OBs and bone-resorbing OCs maintains bone mass. OC function is regulated *via* OB and osteocyte (OCYs) derived RANKL. In the context of metastatic breast cancer disease, breast cancer cells severely disturb the balance between bone formation and resorption through secretion of various growth factors and cytokines [i.e., interleukins (ILs), parathyroid hormone-related protein (PTHrP), matrix metalloproteinases (MMPs), RANKL]. Recently, it has also been suggested that cells from the primary tumor themselves modify the distant microenvironment, for example through systemic factors (i.e., VEGF, TGF-β, G-CSF, miRNAs), in order to make it more attractive for DTCs. Several components of the BME are negatively (red blocks) or positively (green arrows) regulated by miRNAs.

In a physiological context, bone mass is maintained by the balanced activities of matrix-producing osteoblasts (OBs) that originate from mesenchymal cells and can become matrix-entrapped osteocytes (OCYs), and bone-resorbing osteoclasts (OCs) that arise from the hematopoietic lineage (5). OC function and bone resorption is stimulated by the receptor activator of NFκB ligand (RANKL) that is expressed in membrane-bound and soluble forms by OBs and OCYs (Figure 1). The activity is restricted by osteoprotegerin, which is a soluble decoy receptor against RANKL (6). Under pathological conditions, for instance in the context of metastatic breast cancer disease, breast cancer cells colonize the bone marrow microenvironment and severely disturb the balance between bone formation and bone resorption (7). This multi-directional process termed “vicious cycle” perpetuates metastatic bone destruction (8).

MicroRNAs (miRNAs) are small non-coding RNAs and key regulators of various biological processes including bone remodeling and cancer progression (9, 10). miRNAs bind to the 3′UTR of their target mRNAs, and depending on the degree of complementarity interfere with the mRNA stability and/or by block protein translation (9). Abnormal miRNA expression has been implicated in the pathology of osteoporosis, primary bone tumors, and bone metastases of various cancers (11–14). Furthermore, *in vivo* delivery of miRNA mimics or miRNA antagonists has been established as an attractive therapeutic approach to reverse bone degeneration, or to prevent cancer-induced bone diseases (15, 16). Thus, miRNAs can be used as therapeutic targets and may provide a novel tool to treat breast cancer-induced osteolytic disease.

Several miRNAs have been identified to regulate breast cancer cell-intrinsic oncogenic properties, such as proliferation, migration, and invasion (17–19). However, how miRNAs regulate non-cell autonomous interactions in the bone microenvironment (BME) remains largely unknown. This review highlights the recent understanding of the role of miRNAs in the metastatic BME and their potential use as therapeutic targets to modulate the pathological environment and limit disease progression.

## Metastatic Bone Disease

Bone is the most prevalent metastatic site for breast cancer cell colonization and growth. Bone metastasis is a complex multistep process starting from the dissemination of malignant cells into bloodstream, survival of these circulating tumor cells (CTCs) in the circulation, homing to distant organs and eventually metastases formation in the distant site (2). Disseminated tumor cells (DTCs) can be detected in the bone marrow of approximately 30% of breast cancer patients and predict for poor overall survival, breast cancer-specific survival, and disease-free survival compared to patients without DTCs (20).

Once bone metastases occur, the disease is incurable, and treatment remains palliative (21). The standard of care for patients with bone metastases comprises anti-resorptive drugs that reduce the progression of bone destruction and increase survival (22). For instance, bisphosphonates are well established in the treatment of osteolytic disease. Bisphosphonates are incorporated into the bone matrix and taken up by OCs during bone resorption, leading to OC apoptosis and a consecutive reduction of bone resorption (22). An alternative therapeutic approach is the use of the human monoclonal antibody Denosumab (Xgeva^®^) that inhibits RANKL and has been shown to delay the time to first and subsequent SRE in breast cancer patients (23). Although breast cancer patients greatly benefit from the use of bisphosphonates and Denosumab, a better understanding of the control of the “vicious cycle” in the BME and the underlying cellular and molecular mechanisms is needed as it is likely to help identifying novel therapeutic concepts to restrict SREs.

## Tumor Microenvironment (TME)—The BME in Breast Cancer Bone Metastasis

Over the last decade, a variety of preclinical studies have emphasized the contribution of the TME to disease progression (24–28). The TME comprises the cellular environment in which the tumor exists, the surrounding extracellular matrix, and signaling molecules. Several aspects of how the TME impacts cancer growth are well established such as the role of endothelial cells in tumor angiogenesis (29, 30). However, others including the role of the TME in mediating tumor cell invasion, dissemination, and metastasis remain poorly defined (31).

Circulating tumor cells have a high affinity for bone, in particular areas of active bone remodeling (32). The highly balanced cross talk between OBs and OCs, the presence of various other bone marrow-derived cell populations and soluble factors including osteopontin (OPN) and matrix metalloproteinases (MMPs) make bone an attractive site (“soil”) for DTCs (“seeds”). Nearly almost a century ago Steven Pagets’ “seed and soil theory” proposed that therapies to modify the TME might be of equal importance as therapies targeted against the tumor cells themselves (33). Hence, cells of the BME are becoming increasingly recognized as potential therapeutic targets for breast cancer bone metastasis (24–27, 34, 35).

Upon their arrival in bone, DTCs encounter a heterogeneous BME, which is composed of various cells originating from either hematopoietic or mesenchymal stem cells (HSCs, MSCs, respectively) (Figure 1). These include lymphoid and myeloid lineage cells (e.g., immune cells, megakaryocytes, erythrocytes, and macrophages such as OCs) as well as adipocytes and bone and connective tissue-forming cells (e.g., chondrocytes and OBs). In addition, the BME contains a dense, interconnected vascular system which maintains hematopoiesis and osteogenesis (36, 37). Within bone, these various cellular entities form supporting microenvironments, “niches,” which are thought to regulate tumor cell homing, survival, and dormancy (28, 38–41) (Figure 1). The most well-studied niches are the HSC-, endosteal- (OBs, OCs), and vascular niche (vascular endothelial cells, pericytes). Both, the endosteal and the vascular niche control self-renewal, differentiation, and proliferation of HSCs through cell-to-cell contacts as well as by producing a variety of cytokines and intracellular signals (42–45). It is thought that tumor cells respond, similar like HSCs, to these signals. Among the most well studied pathways is the CXCL12/CXCR4 axis. CXCL12 or stromal cell-derived factor-1 (SDF-1) is produced and secreted by bone marrow stromal cells, primarily the OB, endothelial, and epithelial cells (46) (Figure 1). Its cognate receptor CXCR4 is expressed in high levels on various cancer cell lines, including MDA-MB-231 (47). Overexpression of CXCR4 in MDA-MB-231 cells increases bone metastasis, and very recently, it has been demonstrated that both newly and established metastases were anchored in the bone marrow by CXCR4/CXCL12 interactions (48, 49). Further niche signals are suggested to include OPN, vascular adhesion molecule-1, intercellular adhesion molecule-1, chemokines such as Interleukins (ILs) and various growth factors, including bone morphogenetic proteins, Transforming growth factor-β1 (TGF-β), and Vascular endothelial growth factor (VEGF) (50–52) (Figure 1). Emerging data also implicate the importance of the immune- and bone marrow adipocyte niche in bone metastasis (28, 53, 54). Studies by Templeton et al. highlighted the role of adipocytes, one of the most abundant stromal components in the BME, in breast cancer cell osteotropism and early colonization by demonstrating that adipokines, including leptin, promote the migration of MDA-MB-231 breast cancer cells to human bone tissue fragments *in vitro* (28). Nevertheless, exact mechanisms that guide DTCs toward the metastatic site in bone remain to be established. Recently, it was proposed that cells from the primary tumor themselves modify the distant (bone) microenvironment, for example through systemic factors (i.e., VEGF, TGF-β, LOX, G-CSF, miRNAs), in order to make it more attractive for DTCs (27, 55, 56).

Given the heterogeneity of the BME, the fate of DTCs might be determined by the nature of their arrival site within bone. A recent review by Croucher et al. suggests that long-term dormancy might be supported when tumor cells face quiescent/static microenvironments (e.g., endosteal surfaces covered by bone lining cells or stable vasculature), whereas active, dynamic BMEs including areas of osteoclastic bone resorption and sprouting vasculature foster proliferation and/or reactivation of dormant tumor cells (26, 57).

Once activated, breast cancer cells secrete growth factors, such as Parathyroid hormone-related protein (*PTHrP*), ILs, and MMPs, which stimulate OBs to produce excessive amounts of RANKL and other cytokines (58–60). RANKL increases OCs activity and subsequent bone degradation. During bone resorption, matrix-derived growth factors, e.g., TGF-β1 are released into the metastatic microenvironment and further stimulate cancer cell proliferation (7). This “vicious cycle” perpetuates metastatic bone destruction leading to osteolytic disease (8). Therefore, targeting the BME, for instance by miRNAs, to disable this cycle is not only scientifically interesting but also clinically relevant approach.

## Tumor-Derived miRNAs Influencing the BME

MicroRNAs have been recently recognized as key regulators of various biological processes, including cancer progression and metastasis. miRNAs are small (20–22 nucleotides in length), endogenous non-coding RNAs which posttranscriptionally regulate mRNA stability and protein translation (9). More than 1,800 miRNAs are expressed in humans and according to prediction tools, each miRNA regulates numerous target genes (61–63). An important feature of miRNAs is that miRNAs can be encapsulated in extracellular vehicles and released to bloodstream (64–66), which makes them attractive minimal or non-invasive source of biomarkers of various diseases, including bone disorders (67, 68).

MicroRNAs expressed by tumor cells can act as master regulators of bone metastases formation by targeting metastasis-driving factors and consequently altering cancer cell behavior (17–19). In addition, tumor-derived miRNAs can exert their oncogenic or tumor-suppressive action by altering the BME. A specific feature of bone metastatic breast cancer cells is that they exhibit pathologically elevated expression of bone-related genes [e.g., Runt-related transcription factor 2 (Runx2)] and signaling pathways, including the Wnt pathway (69–71). Runx2 is necessary for normal bone formation but often dysregulated in bone metastatic breast cancer cells due to a downregulation of Runx2-targeting miRNAs, including miR-135 and miR-203 (72). Runx2 promotes tumor growth in bone and knocking down Runx2 in cancer cells protects from breast cancer-induced osteolytic disease, positioning Runx2 as an attractive target to reduce bone metastatic burden (73). Indeed, pharmacological delivery of synthetic miR-135 and miR-203 mimics into metastatic breast cancer cells reduces Runx2 protein abundance and consequently, diminishes tumor growth and spontaneous metastasis to bone (72). Furthermore, reconstitution of miR-135 and miR-203 greatly impairs tumor growth in the BME and alleviates osteolytic disease. The bone protecting effect occurred through downregulation of several metastasis-promoting Runx2 target genes, including IL-11, MMP-13, and PTHrP, and subsequent inhibition of OC activity (72).

Similarly, Wnt signaling promotes OB differentiation and function under physiological conditions but a hyper activation of the signaling pathway is implicated in numerous cancers, including metastatic breast cancer (70, 74). In bone metastatic breast cancer cells, Wnt signaling induces the expression of PTHrP thus aggravating the vicious cycle (75). In OBs, Wnt signaling and miR-218 create a positive feed-forward loop through targeting of Wnt inhibitors, such as Dkk1 and sFRP1 by miR-218 (76). Similarly, miR-218 activates Wnt signaling in metastatic breast cancer cells (76). Consequently, miR-218 enhances MDA-MB-231 cell proliferation and increases the expression of Wnt target genes in a Wnt-dependent manner (76, 77). Furthermore, miR-218 promotes PTHrP secretion in cancer cells and subsequent activation of RANKL in OBs, leading to an enhanced OB-mediated OC differentiation. Importantly, antagonizing miR-218 reversed these effects *in vitro* and prevented the formation of cancer-induced osteolytic lesions *in vivo* (77). Tumor-derived osteolytic cytokines are also regulated by miR-211 and miR-379 (78). Both miRNAs prevented TGF-β-induced upregulation of IL-11 and downregulated several genes involved in TGF-β pathway (78). Thus, miR-211 and miR-379 block the vicious cycle by preventing breast cancer cells from receiving signals from the metastatic BME.

Besides regulating the vicious cycle of bone metastasis, tumor-derived miRNAs, including miR-126, have been established in pathological angiogenesis in the BME (79). miR-126, which is silenced in breast cancer cells with bone metastatic potential, suppresses endothelial recruitment and metastatic angiogenesis in a non-cell autonomous manner and, importantly, inhibits bone metastatic colonization of breast cancer cells. The underlying mechanism involves a coordinated targeting of two newly identified pro-metastatic genes; insulin-like growth factor binding protein 2 (IGFPB2) and c-Mer tyrosine kinase (MERTK). Metastatic breast cancer cells secrete IGFPB2 that acts on insulin-like growth factor (IGF1) type I receptor on endothelial cells and modulates IGF1 activation and subsequently endothelial recruitment. In addition, endothelial recruitment is promoted upon cleavage of cMERTK receptor from the breast cancer cells, which antagonizes the binding of GAS6 to endothelial MERTK receptors. A series of elegant loss-of-function and replacement experiments revealed individual components of the pro-angiogenic IGFPB2/IGF1/IGF1R and GAS6-MERTK signaling pathways as direct targets of miR-126 and establish miR-126 as a crucial factor regulating endothelial interactions in the metastatic BME (79).

Recently, miRNAs released from cancer cells in microvesicles or exosomes have been shown to directly control cell–cell interactions in the BME. For instance, miR-192, which is highly abundant in metastatic lung cancer, can be secreted from the cancer cells in extracellular vesicles and transferred to endothelial cells (80). Cancer cell-derived miR-192 is efficiently taken up by endothelial cells *in vitro* and *in vivo*, and inhibits tumor-induced angiogenesis leading to reduced metastatic burden and decreased osteolytic disease in mice.

## Targeting the BME by miRNAs

Since OC activity is a hallmark of metastatic bone disease, the current treatment as well as the majority of basic research is focusing on restricting OC activity and attenuating pathological bone resorption. Along these lines, several studies have established miRNAs as crucial regulators of pathological OC differentiation. Especially miRNAs that suppress bone resorption provide an attractive approach to limit disease progression (81, 82). For instance, miR-34a was recently reported to inhibit physiological and pathological OC differentiation and to block osteoporosis and cancer-induced bone destruction (83). Using several genetic mouse models, Krzezinski et al. demonstrated that OC-targeted overexpression of miR-34a impairs bone resorption resulting in resistance of bone metastases. Conversely, deletion of miR-34a activated OCs leading to reduced bone mass and exacerbated bone metastasis burden. Mechanistically, miR-34a targets a homeodomain protein TG-interacting factor 2, a novel positive regulator of osteoclast differentiation and function. In a therapeutically relevant setting, systemic delivery of miR-34a mimic oligonucleotides *via* chitosan nanoparticles diminished bone metastatic burden and osteolysis (83). Since miR-34a had no direct effect of cancer cell proliferation these effects are likely mediated by osteoclasts and possibly other cells in the BME emphasizing the importance of the TME in disease progression.

In another comprehensive study, a group of miRNAs was shown to regulate tumor-induced osteoclast differentiation. Five miRNAs, miR-33a, miR-133a, miR-141, miR-190, and miR-219 were downregulated during osteoclast differentiation under physiological and pathophysiological conditions (84). Among them, miR-133a, miR-141, and miR-219 impaired osteoclast differentiation *in vitro* by targeting important osteoclast-promoting factors Mitf, Mmp14, Calcitonin receptor, and Traf5. *In vivo* administration of synthetic miR-141 and miR-219 oligonucleotides reduced physiological bone resorption, impaired tumor growth in bone and prevented pathological bone destruction. In this study, two miRNAs, miR-16 and miR-378 secreted in exosomes by osteoclasts were found to be increased in patients with bone metastases compared to healthy controls and the expression correlated with bone metastasis burden (84). Interestingly, miR-378 promotes tumor growth, angiogenesis, and tumor cell survival through the repression of tumor suppressors SuFu and Fus-1 (85). Although beyond this review, it is important to emphasize that miRNA signatures are being pursued as novel clinical diagnostic targets for predicting metastasis or therapeutic resistance (1, 4).

miR-214 is strongly increased in bone specimen of breast cancer patients with osteolytic bone metastases compared to healthy controls and patients without bone involvement (86). Consistently, osteoclasts isolated from mice with bone metastases express significantly higher levels of miR-214 compared to controls. In addition, miR-214 is elevated in bone tissue and serum of aged patients with fractures and miR-214 expression is accompanied with increased osteoclast activity and bone resorption, indicating that miR-214 regulates bone remodeling in health and disease (87, 88). Indeed, miR-214 is expressed and has a functional role in both OBs and osteoclasts. In OBs, miR-214 inhibits differentiation *in vitro* and bone formation *in vivo* by targeting the activating transcription factor 4. As a consequence, delivery of OB-targeted antagomiR-214 in osteoporotic mice increased bone formation and restored bone mass (87). In contrast, in osteoclasts, miR-214 promotes osteoclast function and bone resorption through inhibition of the phosphatase and tensin homolog and Traf3, a negative regulator of NFkB signaling and osteoclast differentiation (86, 89). As a consequence, osteoclast-targeted deletion of miR-214 reduced bone resorption and prevented the development of osteolytic lesions in mice (86). The number and size of non-bone metastases was not changed in mice lacking miR-214 in the osteoclast lineage indicating that the tumor-suppressive effect of bone metastases is mediated by the BME. Importantly, pharmacological delivery of osteoclast targeted (d-Asp8)-liposome conjucated antimiR-214 oligonucleotides reduced physiological and pathological bone resorption and protected from osteolytic bone metastases, suggesting that inhibition of miR-214 could provide an attractive therapeutic strategy to prevent pathological bone destruction (86).

Intriguingly, miR-214 is secreted from osteoclasts in exosomes into circulation and acts on local and distant OBs (88). Treatment of wild-type mice with exosomes isolated from mice with osteoclast-targeted overexpression of miR-214 reduced bone formation demonstrating that the osteoclast-derived miR-214 is fully functional in OBs. This was further supported by an increased bone formation after systemic administration of antagomiR-214 encapsulated in osteoclast-targeted (d-Asp)8-liposomes (88). Given the dual bone anabolic and anti-catabolic effect of antago-miR-214, a systemic delivery of antago-miR-214 might provide a potent strategy to not only prevent osteolytic disease but also reverse existing lesions.

## Perspectives

MicroRNAs play a pivotal role in tissue development and homeostasis. Therefore, dysregulation of individual miRNAs is implicated in several pathological conditions, including the onset and progression of metastatic bone disease. While the role of miRNAs regulating oncogenic properties of tumor cells is relatively well established, the direct or indirect regulation of TME by miRNAs has only recently started to be uncovered. In particular, miRNAs that mediate cell–cell interactions in the BME provide a novel therapeutic approach to disable the pathological cross talk in the bone marrow. For instance, identifying and targeting miRNAs that are pathologically elevated in osteoclasts and promote the vicious cycle could offer novel strategies for diagnosis and treatment of bone metastases. Although the evidence is thus far exclusively based on preclinical data, the applications of using miRNAs as adjuvant tools in bone metastases targets are very promising. Therefore, a better understanding of the complex miRNA-mediated cellular interactions is not only scientifically interesting but also critical in transmitting the knowledge from the bench to bedside.

## Author Contributions

M-TH and HT reviewed literature and wrote the manuscript.

## Conflict of Interest Statement

The authors declare that the research was conducted in the absence of any commercial or financial relationships that could be construed as a potential conflict of interest.
